# Updated ‘N’ Indicators for the National Notifiable Diseases Surveillance System for 2014–2015

**Published:** 2015-06-12

CDC’s National Notifiable Diseases Surveillance System (NNDSS) maintains and annually updates information about which Nationally Notifiable Infectious Conditions (NNICs) are considered “reportable” (i.e., by health care providers, hospitals, laboratories, or other public health reporters) in each of the different reporting jurisdictions. NNICs designated “not reportable” are indicated with an “N”; those conditions that are reportable are recorded with either the number of cases or with a “—” (i.e., a dash symbol) to indicate that no cases were reported for that NNIC. These designations are used in the annual *MMWR* Summary of Notifiable Diseases — United States and in the weekly *MMWR* Notifiable Diseases and Mortality Tables I and II of provisional NNDSS data.

NNDSS staff within the Division of Health Informatics and Surveillance performed assessments with each reporting jurisdiction to ascertain the reportable disease status of each NNIC for the years 2013–2015. The assessment results for 2013 and 2014 were used to populate the “N” indicators for NNDSS data in the *MMWR* Summary of Notifiable Diseases — United States, 2013 and the NNDSS weekly provisional *MMWR* Notifiable Diseases and Mortality Tables I and II for 2014, respectively. Assessment results for 2015 are being used to populate the “N” indicators in the *MMWR* weekly provisional tables for 2015.

When the data for a specified year are reconciled and finalized, NNDSS reporting exceptions (“N” indicators) are summarized by NNIC and reporting jurisdiction in a report that can be found under the Downloads and Resources tab at http://wwwn.cdc.gov/nndss/.

